# Health care needs and barriers to care among the transgender population: a study from western Rajasthan

**DOI:** 10.1186/s12913-024-11010-2

**Published:** 2024-08-26

**Authors:** Tanvi Kaur Ahuja, Akhil Dhanesh Goel, Manoj Kumar Gupta, Nitin Joshi, Annu Choudhary, Swati Suman, Kajal Taluja, Madhukar Mittal, Navdeep Kaur Ghuman, Navratan Suthar, Pankaj Bhardwaj

**Affiliations:** 1 School of Public Health, All India Institute of Medical Sciences, Jodhpur, India; 2 Department of Community Medicine and Family Medicine, All India Institute of Medical Sciences, Jodhpur, India; 3 Department of Endocrinology and Metabolism, All India Institute of Medical Sciences, Jodhpur, India; 4 Department of Obstetrics and Gynaecology, All India Institute of Medical Sciences, Jodhpur, India; 5 Department of Psychiatry, All India Institute of Medical Sciences, Jodhpur, India

**Keywords:** Transgender persons, Gender dysphoria, Focus groups, Depression, Mental Health, Non-communicable diseases, Health services

## Abstract

Transgender people comprise an estimated 0.3–0.5% (25 million) of the global population. The public health agenda focuses on understanding and improving the health and well-being of gender minorities. Transgender (TG) persons often have complex healthcare needs and suffer significant health disparities in multiple arenas. The international literature suggests that this community is at a higher risk of depression, and other mental health problems, including HIV. Many transgender people experience gender dysphoria and seek specific medical needs such as sex reassignment surgeries, implants, hormonal therapies, etc., but are unable to access these services due to financial or social reasons. The objective of this study was to assess the healthcare needs and associated barriers experienced by transgender people in Western Rajasthan. Methodology: A qualitative study was carried out in which multilevel stakeholder interviews were conducted using interview and focus group discussion guides. Data was analyzed using the qualitative thematic analysis technique. Results: Findings reveal that transgender people have expressed their need to access health services for general health needs, including but not limited to mental health, non-communicable diseases, and infectious diseases. Barriers to healthcare services were identified on 3 levels: health system, social and personal. Health system barriers include policy, accessibility, affordability, and acceptability issues. Social factors such as inadequate housing, education, and job opportunities also play an important role in affecting the individual’s health-seeking behavior. The knowledge of healthcare providers in this context was also limited in context of health insurance schemes, package of services available for transgenders and the importance of gender sensitive healthcare. Conclusion: Transgender people expressed the need for mental health services, programs targeting nutritional improvement, gender-affirmation procedures besides regular screening of non-communicable diseases as operational for males and females. Levels of barriers have been identified at various levels ranging from absence of targeted policies to individual behavior.

**Supplementary Information:**

The online version contains supplementary material available at 10.1186/s12913-024-11010-2.

## Introduction

Universal Health Coverage (UHC) means that all people have access to quality healthcare services (service coverage) without any financial hardships (catastrophic health expenditure) [1]. To achieve UHC, National Health Policy 2017 projects to increase the government’s health expenditure to 2.5% of GDP by 2025 [2]. In alignment with this objective, the budgeted health sector spending has increased from 1.3% (2019-20) to 2.1% of GDP (2021–2022). According to National Health Accounts 2018-19, Out of Pocket Expenditure per capita (in Rupees) is 2155 [3].

Incidence of catastrophic health spending is felt at a higher rate by vulnerable communities due to gender, socio-economic position, disability status, or sexual orientation, besides other characteristics. The Transgender (TG) community is one such community whose gender expression (masculine, feminine, other) differs from their assigned sex (male, female) at birth. They can be identified as trans-man or trans-woman [4]. In the Indian context, transgender individuals identify themselves differently including Hijras, Aravanis, Kothis, Jogtas/Jogappas, and Shiv-Shakthis. For the first time, this population was included in India’s 2011 census. Reports suggest that 4.8 million Indians identified themselves in the ‘other’ category.

Recent legal and policy changes in India have significantly affected gender-diverse communities. The Transgender Persons (Protection of Rights) Bill formulated in 2014 went through changes over a period of 5 years and was finally declared an Act in 2019. The Act highlighted the need to prohibit discrimination including denial of service or unfair treatment in relation to healthcare [5]. With respect to UHC, there are evident disparities in service coverage across the transgender population [6]. Transgender persons face a disproportionate burden of certain diseases, including HIV, viral hepatitis, and other sexually transmitted infections. They also pose a higher risk for mental health issues or substance abuse. Literature also suggests that transgenders may seek gender-affirming health services apart from general healthcare services. This might involve counseling support for themselves and their family related to gender id entity or undergoing gender transitioning procedures and surgeries. Transition-related treatment may include cross-sex hormonal therapy, hair removal, and gender-affirming surgeries.

In India, there are significant disparities in the availability and accessibility of healthcare. Existing research has identified various challenges and barriers encountered by transgenders in accessing and navigating the healthcare system. These include a lack of provider expertise in transgender care, the gap in health systems delivery mechanisms, lack of culturally sensitive healthcare training, inadequate financial coverage or low socio-economic condition, and poor community health-seeking behavior [7]. Rajasthan is one of the high focus states under the National Health Mission [8]. Since Western Rajasthan is a desert area, healthcare becomes even more challenging [9]. It further causes adverse impact on the desire and ability of transgender people to access healthcare. Poor healthcare access and health outcomes among the transgender population can also be attributed to lower levels of health literacy [10]. Rajasthan is one of the states with the lowest literacy rates among the transgender population [11].

In order to improve the health of transgenders and address the barriers to healthcare, it is crucial to identify the health priorities. A growing body of research regarding the healthcare experiences of transgenders exists worldwide, but there is still a paucity of research in the Indian context. This study has been conducted with the aim of reviewing the health issues and challenges faced by them in the existing healthcare system in Western Rajasthan. Although behavioral and social factors play a pivotal role in transgender health, this research focused on health needs and healthcare system-related barriers and challenges.

Research questions:

- What are the basic health needs of the transgender community?
- What are the barriers they encounter in the process of obtaining healthcare services?
- How are the experiences of transgender persons in healthcare facilities?
- What is the level of knowledge among healthcare providers regarding health of transgender persons?
- How can healthcare services be enhanced for the transgender population?

## Methodology

The study was conducted in the state of Rajasthan during the year 2022.

Research Design: This study utilizes a descriptive qualitative research design to allow in-depth insight into the existing health-related needs of transgender persons, their experiences in healthcare facilities and the barriers they encounter in meeting their needs. The study was approved by the Institutional Review Board of All India Institute of Medical Sciences, Jodhpur in July 2022.

Setting: The study was conducted in a community-based organization.

Sampling and sample size: Purposive sampling was utilized for this research. Transgender people above or equal to 18 years of age residing in different geographical region of western Rajasthan were approached with the help of established Civil Service Organizations (CSO). People who responded back were included in the study.

Data collection: All recruitment and data collection procedures were completed by public health scholars trained in research under the supervision of a community medicine professor. Multi-stakeholder interviews were conducted. It includes identification of relevant stakeholders to understand facilitators and barriers of the topic of interest. The identified stakeholders included specialist healthcare providers, representatives of civil service organizations and transgender persons (target population). All the individuals who agreed to participate in the study were approached by the interviewer. They were explained the purpose of the study and an appropriate time was decided to ensure active participation. This was also done keeping in view the sensitivity of the subject matter. All the interviews and the focus group discussion were conducted face-to-face in English and translated to Hindi for participants who did not understand English. Key informant interviews (*n* = 7) of specialist healthcare providers central to providing transgender care were conducted. The specialists included of a psychiatrist, plastic surgeon, endocrinologist, gynecologist, and community medicine experts. All interviews with specialist healthcare providers had a duration of 20–30 min. Transgenders residing in different geographical region of western Rajasthan were invited for a focus group discussion (FGD). Informed consent was taken from all the study participants who agreed to participate in the study. Audio and video recordings were done for focus group discussion as well as key informant interviews. The focus group discussion lasted for 2 h. Apart from this, CSO representatives were also interviewed (*n* = 3) i.e., a nurse, social worker and the administrative head of the organization. Data was collected over a period of 2 months.

Before the focus group, socio-demographic data was recorded, including age, gender, education, and income. An FGD guide and interview schedules were prepared and used for focus group and key informant interviews, respectively. They were designed to cover information about:

1. Knowledge and experience on transgender issues.
2. Challenges in providing transgender care.
3. Methods for improvement of the healthcare system.

Analytic approach: Audio-recorded focus group data and key informant interviews were transcribed and translated into English by four researchers. The data obtained through key informant interviews were also transcribed. The data was analyzed manually using thematic analysis. The available data was actively and repeatedly read to familiarize and valuably orient towards the available raw data. Subsequently, codes were identified using an inductive approach i.e., they were reflective of the issues that were apparent in the data and were not dependent or guided by any existing theoretical frameworks. In the next step, themes were constructed by analyzing, combining, and comparing codes. The developed themes were such that they reflected the significance of the entire dataset. Lastly, the themes were reviewed, defined, and named, along with the identification of narratives that justify and explain all the mentioned themes. In the final stage of analysis, the identified themes from the coded data were used to construct a framework using grounded theory approach such that it accurately represents a concise picture of the data.

Ethical considerations: Confidentiality emerged as an ethical concern in this study. All transgender individuals were provided with detailed information about the purpose, procedures, potential risks, and benefits of research. Participants were ensured that their participation was voluntary, and they had the right to withdraw at any time without consequence. All data and identifying information collected during the discussion was restricted to the research team and anonymized to prevent identification of individual participants.

## Results

A total of 12 transgenders participated in the FGD. Their socio-demographic characteristics are summarized in Table 1. All Participants in the study belonged to Rajasthan, India. Eleven out of the total 12 participants self-identified themselves as transgender woman. The mean age of transgender individuals who participated in the study was 23.8 ± 3.6. The selected cohort represented a range of educational qualifications from secondary school to post-graduation. The majority of the participants were employed, but none was employed in the government sector. More than half of the participating individuals had an income of less than INR 10,000 (66.7%) (Table 1).

**Table 1:** Socio-demographic profile of transgender participants (*n* = 12)

SOCIO-DEMOGRAPHIC PROFILE	
Age (mean ± SD, in years)	23.8 ± 3.6
Gender identity	
Transwoman (Male to Female) Transman (Female to Male)	11 (91.7) 1 (8.3)
Education	
Secondary (6th – 11^th)^ High School Graduation Post-Graduation	5 (41.6) 2 (16.6) 3 (25.0) 2 (16.7)
Employment	
Employed Unemployed	11 (91.7) 1 (8.3)
Monthly Income	
<10,000 >10,000	8 (66.7) 4 (33.3)

*Numbers in brackets represent column-wise percentages

### Health needs of the transgender community

The need for regular screening of non-communicable diseases at peripheral healthcare centers was expressed by the transgender participants. Lack of accessible and/or affordable health services and social barriers contribute to anxiety and depression among them, which further leads to their inability to control the use of tobacco and alcohol. This indicates the need for mental health support tailored specifically for this population.

**Healthcare provider 1 (HCP-1)**

“*Gender dysphoria is diagnosed in later stages of life, late adolescence, or early adulthood because individuals are not able to seek help due to a lack of knowledge on available medical options and familial pressure.”*

Specifically, they expressed the need for public healthcare facilities to provide gender transitioning procedures ranging from hormone replacement therapies to sex reassignment surgeries.

**Fig. 1:**
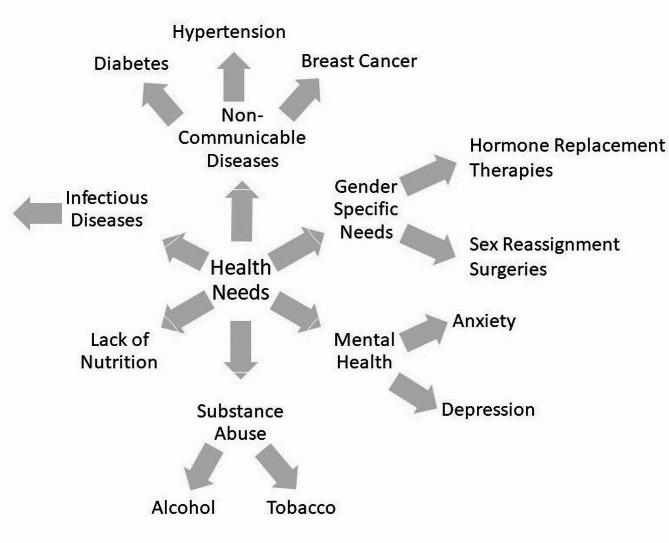
Coding tree for health needs of transgender participants

Figure 1 illustrates coding tree for health needs of transgender participants.

Barriers enumerated by transgenders in accessing healthcare services were segregated into personal, healthcare system and social barriers (Table 2).

**Table 2:** Barriers faced by transgender participants in seeking healthcare

LEVEL OF BARRIER	THEMES	CODES	VERBATIMS/NARRATIVES
**PERSONAL**	Health Seeking Behavior	Lack of awareness regarding quality healthcare services	*‘Some people go to unqualified or traditional medical practitioners for surgery, sometimes they also die in that case’*
**HEALTH SYSTEM**	Policy	Health Insurance Diverse Gender Policy	*‘Whenever we go to the hospital, we are asked whether to write male or female’* *‘I had fever since a few days, I went to a hospital for treatment. I gave my transgender ID card issued by the ministry. They said this is not valid’*
	Accessibility	Long waiting time Long counselling time for HRT Unprescribed hormonal treatment Distance to health facilities Treatment by traditional practitioners/ Dais	*‘I accompanied my friend to a hospital where she had to consult a gynecologist. We kept waiting outside for a long time. After 1 hour, when we angrily told the staff, then they let us in.’* *‘We are already very upset and due to these long and time-consuming counselling sessions; we start self-administered doses of hormones’*
	Availability	Untrained healthcare providers Lack of transgender care at primary level Lack of specialist care Dedicated healthcare facilities	*‘Whenever we go to the hospital, there are only male and female queues. Where should we stand? Who will do our checkup? Male or female doctor? Separate beds are not assigned for us; therefore, we approach the Unani doctors near our homes.’*
	Affordability	Financial strain	*‘Some people go to unqualified or traditional medical practitioners for surgery, sometimes they also die in that case*
	Acceptability	Stigma Discrimination Lack of social support	*‘Everybody looks at us in such a weird way; we feel uncomfortable.’* *‘People should accept our community; we are also a part of it. We are not downloaded. Like the government promotes the use of condoms, posters should be displayed even for our acceptance.’*
**SOCIAL**	Environment	Unemployment Poor housing conditions Food insecurity	*‘People of the community are not able to find housing in good localities, even the washroom are not clean.’*

### Personal barriers

Transgender participants revealed a lack of awareness regarding the provision of transgender identity cards being issued by the Ministry of Social Justice and Empowerment. Moreover, the growing need was identified to educate them regarding their entitlements which may have implications on health. These include but are not limited to recognition of their gender identity, provision of medical facilities for their surgical and hormonal needs, and facilitation of access in hospitals and other healthcare facilities. (Transgender Act 2019)

During a key informant interview, one of the medical practitioners highlighted the need to introduce and explain the range of medical options available to transgenders for their transition.

**HCP-2**

“*Internationally, I have worked in fertility clinics. Before undergoing hormonal therapies or surgeries, transgender patients usually preserve oocytes and sperms to bear children in the future. The basket of available options must be known to the community. This also improves their quality of life.”*

The health outcomes of an individual are dependent on their timely health-seeking behaviors. An interview revealed that many transgenders prefer the traditional removal method of male genitals rather than conventional gender affirming surgery. This reflects multiple dimensions such as lack of awareness regarding appropriate health practitioners and discrimination by the qualified professionals. Other underlying reasons for this include the lack of public hospitals providing these services and the unaffordable costs of surgeries. A study participant has also revealed being comfortable getting the surgery done by the *‘guru’*. Moreover, the distance between their households and healthcare facility makes it inaccessible for them.

### Health system barriers

#### Policy

Both transgender persons and healthcare providers reported a lack of knowledge of any insurance schemes specifically for transgenders or insurance coverage for the minority population under the available schemes. Their awareness regarding the inclusion of gender-specific needs such as sexual reassignment surgeries or hormonal therapies in the existing insurance schemes was limited.

The study participants also addressed the need for the inclusion of a third gender column in the patient information / outpatient cards across all the hospitals. This is in alignment with the Transgender Persons (Protection of Rights) Bill, 2019, which prohibits discrimination against them in healthcare [5]. It would also lead to a transgender-inclusive environment in the hospital and greater acceptance by other people.

**TG participant 7**

“*Whenever we go to the hospital, we are asked whether to write male or female. There is no option of transgender in the OPD cards.”*

In India, nationally recognized identity cards are being provided by the Ministry of Social Justice and Empowerment as a step towards mainstreaming their identity. One participant revealed that recently when she visited a hospital, the authorities denied accepting the TG identity card. This incident reflects the need of generating awareness across all sectors, including healthcare, to prevent the exclusion of transgender people in society.

**TG participant 8**

*I had fever for a few days, I went to a hospital for treatment. I gave my transgender ID card issued by the ministry. They said this is not valid.*

#### Accessibility

Majority of the participants revealed having negative experiences in healthcare settings. They reported that they had to wait very long to access health services.

One participant complained about the long counselling procedure and time to access hormone replacement therapy. Furthermore, many qualified practitioners discourage and demotivate the use of hormones. This reluctance among medical practitioners to prescribe hormones often compels transgenders to refer to the unfiltered content on the internet, resulting in the self-administration of hormones. Since transgenders are unaware of the side effects of unregulated dosages of hormones, it can result in adverse health outcomes.

Sometimes, the health facilities with available resources are situated far away from the residence of transgenders leading to difficulty in access. In one of the key informant interviews, a medical practitioner shared her experience with a transgender patient whose vaginal canal got stenosed as a complication of post Sex Reassignment Surgery (SRS). Since the health facility was around 500 km from her hometown, she could not reach the hospital on time.

#### Availability

One of the most significant barriers to healthcare reported by transgenders was a dearth of healthcare providers trained to address their specific health problems. Healthcare providers also emphasized the need for training to understand the best practices for their care. Some parts of clinical training should also include the importance and impact of physician-patient communication. The use of correct pronouns should be taught to collect sufficient and accurate information on their gender identity and thus, making the hospital settings friendly for them. Additionally, awareness sessions should also be conducted for medical professionals to make them comfortable and culturally competent while dealing with this section of society.

Some participants also shared that there is a need for designated facilities in healthcare, such as separate queues in OPDs and dedicated wards or beds in hospitals. It was felt that these facilities’ absence contributed to their fear and delay in access and utilization of desired appropriate care. Due to contributory social factors, such as real or perceived stigma, it is challenging for them to accommodate within the general ward. Medical providers had contrasting views in lieu of the unavailability of designated facilities. While most believed that providing separate queues and beds for them in hospitals was essential, one of the doctors felt this would promote social exclusion.

**TG participant 1**

*Where should we stand in hospitals? Queues made for males or females? Separate beds shall be assigned for us so that we can access the services without hesitancy or fear of discrimination*.

**HCP-3**

*Providing them separate facilities for all services cannot be the ultimate solution. Will this promote equity or rather advance social exclusion? We should think about it.*

Moreover, there is a lack of specialist care in hospitals that are accessible to them. There is no provision to address transgender-specific health problems at the primary healthcare level. Lack of robust referral mechanisms leading to delayed or denied care was also reported.

#### Affordability

Transgenders are not registered and do not have access to benefits under the insurance schemes functioning in the country. All hospitals in the country do not provide gender transition services. Those services provided by the private sector often have charges beyond their paying capacity. As a result, accessing and affording healthcare becomes a challenge for them. This is one reason that urges them to go to unqualified traditional medical practitioners for gender transitioning surgeries or *‘Dai Nirwan.’*

Breast augmentation is another common procedure utilized by transgenders. One participant discussed the availability of various implant materials and how their costs vary depending on the quality. Additionally, due to financial reasons and lack of awareness, low-quality implant materials are utilized in surgeries, which increases their risk for breast cancer.

### Social barriers

The non-medical factors play a crucial role in impacting health outcomes. Addressing social determinants is central to reducing existing health inequities. In this study, all the participants reported stigma and discrimination while sharing their experiences in healthcare settings. They further added that this discouraged them from utilizing available health services.

The participants reported that even the healthcare providers were uncomfortable with their presence and did not treat them like other patients.

Poor housing conditions and lack of job opportunities further push them into this vicious cycle of stigma and sickness. Transgenders have also reported experiencing psychological distress due to a lack of social support. Positive attitude and gender-supportive relationships in society can promote their well-being. The need for their inclusion in society through awareness generation by government initiatives was emphasized.

**TG participant 3**

*We can promote family planning through condom advertisements, so why not involve transgender figures in government health awareness advertisements and campaigns*.

### Healthcare provider expertise in TG health

All the healthcare providers felt the need for training to improve physician-patient communication and transgender persons care. A culturally competent healthcare perspective is fundamental for treating the transgender population. Those providers who had experience with such patients were more likely to provide perspectives on their care and barriers than those who had never encountered such cases. They highlighted that very few transgender patients are registered in the hospitals of Rajasthan. This can be attributed to the stigma associated with their presence rather than assuming they do not wish to seek healthcare services.

## Discussion

This study sought to investigate and fill the gap in the domain of transgender healthcare. The purpose of the research was to characterize the health needs and barriers faced by transgender individuals in navigating through the health system. Previous international and Indian studies have reported a lack of transgender-sensitive care. The findings of this research corroborate this premise. There is a wide and serious gap between the population’s needs and the healthcare system’s ability to respond to these needs.

The socio-demographic profile of the participants in this study revealed that the income of the majority of the participants was below INR 10,000. This finding is in alignment with the results (70%) of a study conducted among transgenders in Vadodara, Gujarat, India [12].

The FGD gave an opportunity to the study participants to express their general and gender-specific health needs. The health needs of the participants in this study included available medical services common to the general population and certain specific transgender needs, particularly psychiatric support, hormonal therapies, and sex-reassignment surgeries. This is in accordance with the previous studies, which also identified general health problems that need to be addressed, including the high prevalence of diabetes and hypertension, substance abuse, anxiety, and depression [13].

Transgender individuals discussed a range of experiences and barriers encountered in the healthcare system in accessing the available services. The barriers were categorized at the healthcare system, social and individual levels. The system-level barriers ranged from policy issues to hospital or organizational problems. It included a lack of coverage for the transgender population in government health insurance schemes. The introduction of a comprehensive package master in the Ayushman Bharat scheme has now addressed the lack of coverage for transgenders in the existing insurance schemes. It includes the existing packages as well as specific packages for transgenders [14]. This paves the way for a new chapter in their care. The unavailability of trained healthcare providers is another major problem. In 2019, National Medical Commission (NMC) updated the medical education curriculum and added a new module on Attitude, Ethics, and Communication (AETCOM) competencies [15]. It could be used as an opportunity to introduce culturally sensitive communication training for medical professionals, especially focusing on LGBTQ + community, to advance our aim to achieve equity. In addition to the unavailability of trained doctors, the inaccessibility of healthcare facilities and unaffordability also negatively impact the people’s health. All these underlying factors contribute to their practice of getting surgeries done by traditional and untrained medical practitioners. These findings are consistent with another study conducted in India to assess the health-seeking behavior of transgender people. They also reported long waiting times in hospitals affecting their health behaviors and are confirmed to have undergone medical procedures performed by gurus or technicians [16].

The finding of concern that emerged in our study sample was the use of unprescribed hormone therapy. This finding is similar to a study conducted in Maharashtra to assess the practices related to hormonal therapy [17]. It states that participants reported going for unsupervised hormone replacement therapy due to unaffordability, lack of trained healthcare providers and prior experiences in healthcare settings [17]. In order to avail the hormonal therapy, transgender patients require to undergo psychological counseling’s for confirmation of gender dysphoria. In our study, transgender individuals felt that the psychotherapy sessions are too long, leading to a delay in the initiation of hormone replacement therapy. World Professional Association for Transgender Health (WPATH) mentioned in their Standards of Care (SOC) that any minimum number of sessions cannot be fixed and is an individualistic approach. It depends whether someone wishes to avail psychological support before, during, or throughout the transition process [18]. Gender transitioning may involve but not be limited to procedures such as hormonal therapy and sex reassignment surgeries. Moreover, there are only a few public health facilities providing gender-transition services and there is no government support in the form of subsidies to avail these services from a private hospital. The government, is however, working on extending and empaneling public and private hospitals in order to make these services accessible to the population.

This need assessment study also attempted to address the social determinants barring healthcare access. Stigma, discrimination, support from family and friends, and difficulty in seeking housing determine health and healthcare accessibility. These factors have also been highlighted by the study conducted in Vadodara, India [12]. It re-emphasizes the findings from our study that social determinants such as lack of economic and educational opportunities, rejection, and isolation from society have an impact beyond gender identity issues, rather, they pose a risk to the psychological status of the transgender population.

The health disparities and barriers to care faced by transgenders should be addressed to promote health equity and justice. Comprehensive approaches to improve access, utilization, and quality of healthcare services are currently lacking. These challenges can also be addressed at the following levels:

- Individual Level.
- Healthcare system Level.
- Community Level.

*Garima Greh* facilities have been introduced as shelter homes for transgender individuals where basic amenities are being provided to them [19]. They can be utilized as launchpad sites to improve their awareness of their rights and available entitlements and medical interventions. IEC materials can be displayed at Garima Greh facilities for health promotion and modification of their health-seeking behaviors. Similar to ASHA workers who are community members working for their healthcare, volunteers can be appointed from their community. Training of these volunteers can be done (Training of Trainers) so that they can improve their health-seeking behavior, increase awareness, and aid in the overall empowerment of the community.

All hospitals and clinical settings shall provide a safe and welcoming environment for gender-diverse people [20]. The fact that their physical, mental, and cultural differences affect their behaviors must be known but, more importantly, understand these differences and assigning them value is the key. Actions can be taken to promote transgender identity across healthcare settings by displaying Information, Education and Communication (IEC) material regarding their health needs and promoting their acceptance in society – ‘This hospital is LGBTQIA + friendly.’ Transgenders can also be part of the healthcare system, whereby; they can act as resource persons and promote the inclusivity of gender-diverse individuals. Medical students shall be trained to communicate sensitively to the needs of transgenders, and doctors shall be trained in a culturally competent way to treat their gender-specific needs. To promote access and utilization of health services, specific transgender clinics are being set up across the country. All participants in the study felt this would help them to access available services without hesitancy. Separate general health camps for regular and dental check-ups can also aid in health promotion and equity. Another potential solution to promote transgender health is through digital solutions. Tele-consultation can be an effective way to address their needs as well as to protect them from social stigma and discrimination that hinder their access and utilization of available services.

According to World Health Organization (WHO), social determinants account for 30–55% of health outcomes [21]. The most effective way to address SDH is by action at the community level. General campaigns and community awareness sessions are essential to promote acceptance by the general population. Moreover, the study suggests that there is a need for awareness and sensitization of transgenders regarding the basket of medical options available for them such as techniques for fertility preservation.

In summary, the study demonstrates the health issues of transgenders and reflects upon the various factors influencing health and access to care. It urges the stakeholders to contemplate the need to safeguard the rights of transgenders by providing equitable access to the available resources.

This study is an attempt to explore health needs from beneficiary as well as service provider perspectives. Our findings are consistent with the previous literature. Findings from this study provide evidence base for future research and a helpful tool for the policymakers and advocates to better address the needs of transgender people.

The study’s major limitation was that only one focus group discussion was undertaken due to limited time and difficulty in accessing the desired population. However, one FGD allowed for exploration of issues related to transgender experiences and healthcare needs. It allowed the researchers to gather detailed narratives that might not emerge from individual interviews. Given the paucity of literature in the Western Indian context, a single focus group discussion can be valuable for informing advocacy efforts and policy reforms. Participants in the study were recruited through purposive sampling and did not differentiate between cultural identities of transgender persons; therefore, the results might vary geographically and according to the social context, thus, limiting the external validity. The health needs might vary between transgender male and female populations, but there was only one transgender male participant in our study. Additionally, the service providers’ knowledge was not directly assessed by explicitly questioning the standards of care.

### Proposed framework

**Fig. 2:**
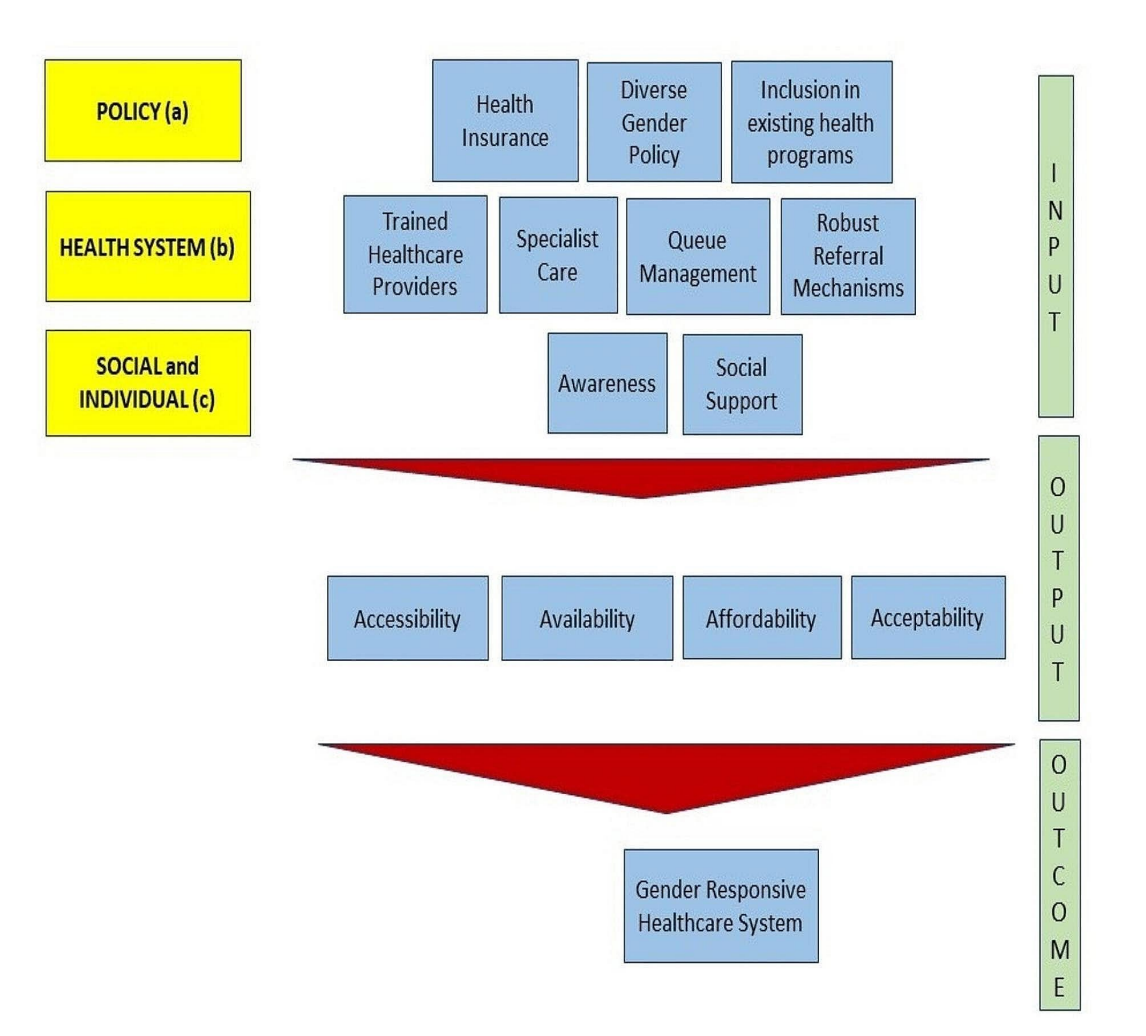
Gender Responsive Healthcare System Framework

Based on study findings, a Gender Responsive Healthcare System Framework is designed. (Fig. 2) This framework illustrates and emphasizes on the need for planning, interventions, and actions at 3 levels – policy (a), health system (b), social, and individual (c) in alignment with the identified themes represented in Table 1. The framework describes how the barriers can be addressed at these 3 levels to have a robust and gender-responsive healthcare delivery system in India.

The concept of healthcare is multi-dimensional. Combined action is required at the administrative, service provider and beneficiary level for a gender responsive healthcare system. Inclusion of the transgender population in existing health insurance schemes is central to reducing their out-of-pocket expenditure and helping them gain recognition in the society through the treatments that they wish to access. Outpatient cards in hospitals should include options of male, female and transgenders/others (gender diverse) creating a safe and welcoming environment. Existing health programs shall also target transgender population for reducing the burden of infectious diseases such as tuberculosis and non-communicable diseases. At the healthcare system level, medical professionals competent to provide transgender specific care and availability of specialists shall be ensured. A robust referral mechanism from primary healthcare centers to higher levels could ensure uninterrupted care for the transgender population. Moreover, hospitals have distinct queues, for men and women. There is a need to understand that gender is not a visibly readable or unchanging phenomenon, rather it is a social construct. Proper queue management can address not only the issues of stigma, but also make healthcare accessible to them. At an individual level, good health seeking behavior and familial support can aid in improving health outcomes. Altogether, these efforts at the policy, health system and individual level can lead to improvement in accessibility, availability, affordability and acceptability of services by the transgender people.

## Conclusion

This study has explored experiences of transgender people navigating through the healthcare system. These accounts have highlighted their health needs and the barriers they face in accessing care. They expressed the need for mental health services, programs targeting nutritional improvement, gender-affirmation procedures besides regular screening of non-communicable diseases as operational for males and females. Levels of barriers have been identified ranging from absence of targeted policies to individual behavior. Targeted efforts and intersectoral collaboration are required for effective establishment and delivery of healthcare services.

### Electronic supplementary material

Below is the link to the electronic supplementary material.

Supplementary Material 1

## Data Availability

The data generated and reviewed are fully available in this article and its supplementary files. For any further data, Dr. Tanvi Kaur Ahuja (drtanvikaur.nhsrc@gmail.com could be contacted).

## Data availability

All relevant data analyzed during this study are included in this published article and its Supplementary Information files.
