# Supplementary material for: Health care needs and barriers to care among the transgender population: a study from western Rajasthan
Source: BMC Health Serv Res. 2024 Aug 26;24:989. doi: 10.1186/s12913-024-11010-2 (PMC11348736; doi:10.1186/s12913-024-11010-2)
Supplement: Supplementary file 1 — Supplementary Material 1 [file 12913_2024_11010_MOESM1_ESM.pdf]

Supplementary Material

Healthcare providers interview guide

1. What healthcare problems do you commonly encounter in this community
2. From your experience, what are the transgender specific needs that should be addressed?
3. Do you think a policy is required for stigma and discrimination free environment for transgenders?
4. Experience with any insurance scheme for transgenders?
5. Based on your experience, how the government systems must be strengthened for providing healthcare services to this transgender community?
6. Do you suggest any trainings for medical or paramedical staff for addressing health needs of transgenders? Any previous trainings attended by you?

Focus Group Discussion Guide

1. What health problems do you face in your daily life?
2. Any specific health needs that you wish to address?
3. Have you ever visited a hospital? Share your experience in healthcare settings
4. How do you think we can improve healthcare service delivery for transgenders?
